# 生物亲和垂钓技术在天然产物活性成分发现中的研究进展

**DOI:** 10.3724/SP.J.1123.2025.08021

**Published:** 2026-08-08

**Authors:** Qingli QU, Xiaofang WANG, Duolong DI, Dong PEI

**Affiliations:** 1. 中国科学院兰州化学物理研究所，中国科学院西北特色植物资源化学重点实验室和甘肃省天然药物重点 实验室，甘肃 兰州 730000; 1. CAS Key Laboratory of Chemistry of Northwestern Plant Resources and Key Laboratory for Natural Medicine of Gansu Province，Lanzhou Institute of Chemical Physics，Chinese Academy of Sciences，Lanzhou 730000，China; 2. 青岛市资源化学与新材料研究中心，山东 青岛 266000; 2. Qingdao Center of Resource Chemistry and New Materials，Qingdao 266000，China; 3. 甘肃药业集团科技创新研究院有限公司，甘肃 兰州 730000; 3. Gansu Pharmaceutical Group Technology Innovation Research Institute Co. ，Ltd. ，Lanzhou 730000，China

**Keywords:** 天然产物, 生物亲和垂钓技术, 固定化, 载体, natural products, bioaffinity fishing technology, immobilization, carriers

## Abstract

天然产物是创新药物发现的重要源泉，其活性成分的高效、精准筛选是推动天然药物研发的关键。传统筛选方法步骤烦琐、周期长，而基于表型的高通量技术又存在作用机制不明确的局限性。近年来，以生物亲和垂钓为代表的靶向筛选技术因其能够将复杂体系中的分离与活性筛选相结合，实现了从天然产物中直接、快速“垂钓”特定靶标的配体分子，而受到广泛关注。本综述聚焦于生物亲和垂钓技术的核心——垂钓材料，从载体材料和固定化方法两个维度系统梳理了近3年的研究进展。在载体材料方面，文章详尽阐述了从无载体（如亲和超滤、交联酶聚集体）到有载体材料体系，后者包括磁珠、金属有机框架、硅基材料、凝胶、微通道、荧光材料等各类新兴材料的设计、优势及其应用实例。在固定化方法方面，文章对比分析了物理吸附、物理包埋与化学交联3种策略的特点与挑战，并重点探讨了基于生物正交反应和非天然氨基酸插入等定向固定化策略在保持靶标活性与结构完整性、提高固定效率与色谱性能方面的突破性进展。文章最后展望了该领域的未来发展方向，指出开发兼具高亲和性、高稳定性及低非特异性吸附的多功能复合材料，优化定向固定化策略以适用于内源性靶标，以及整合实时活性监测功能（如荧光材料）是实现更高效、可靠的一站式筛选平台的关键。生物亲和垂钓技术作为一种强大的工具，将继续在阐明天然药物药效物质基础及加速创新药物发现进程中发挥不可或缺的作用。

## Abstract (en)

Natural products have consistently served as an invaluable source for novel drug development， and the efficient and targeted screening of bioactive constituents from these complex mixtures is crucial for advancing drug discovery. Bioaffinity fishing techniques exploit the highly specific interactions between ligands and their biological targets—such as antigen-antibody， enzyme-substrate， and hormone-receptor interactions—to simultaneously separate and identify active compounds from complex natural product extracts. When coupled with liquid chromatography-mass spectrometry， bioaffinity fishing enables the integrated separation， screening， and analysis of bioactive molecules in a single step. This comprehensive review focuses specifically on bioaffinity fishing materials， which are fundamental to the effectiveness and reproducibility of the fishing process. The review systematically explores recent advances over the past three years， categorizing the discussion along two primary dimensions： carrier materials and immobilization methods. In terms of carrier materials， the article provides a comprehensive review of both carrier-free and carrier-based systems. Carrier-free approaches， including affinity ultrafiltration and size exclusion chromatography， utilize free targets in solution， while carrier-based systems immobilize the target onto a solid support. The review details a wide spectrum of innovative supports such as magnetic beads， which facilitate easy separation； metal-organic frameworks， known for their high surface area and tunable porosity； silica-based materials （e.g.， mesoporous silica and silica chromatographic stationary phases）， prized for their stability and versatility； gel-based materials like agarose and synthetic polymers， which offer superior biocompatibility； microchannel-based systems， including capillary columns and microfluidic chips that enable high-throughput screening with minimal sample consumption； fluorescent materials that integrate fishing with real-time activity monitoring and imaging； and other emerging materials such as graphene， carbon nanotubes， and paper-based substrates. Each material system is evaluated regarding its preparation， advantages， limitations， and representative applications in fishing active ingredients from natural products. Regarding immobilization strategies， the review compares three principal methodologies： physical adsorption， which is simple but often prone to leakage and low efficiency； physical encapsulation， which protects the target yet may incur mass transfer limitations and potential activity loss； and chemical cross-linking， which enhances stability but risks altering the target’s conformation and activity. A significant portion of the discussion is devoted to advanced directional immobilization techniques designed to preserve biological activity and improve efficiency. These include various bioorthogonal reactions such as Halo-tag/alkyl chloride， His-tag/Ni-NTA， SpyTag/SpyCatcher， SNAP-tag/benzylguanine， and the use of unnatural amino acid incorporation combined with click chemistry （e.g.， azide-alkyne cycloaddition， thiol-ene addition）. These strategies allow for precise orientation and covalent attachment of proteins， leading to improved stability， reduced nonspecific binding， enhanced chromatographic performance， and higher binding affinity. Notably， these methods enable the one-step immobilization of even low-abundance or endogenous targets without the need for prior purification， greatly expanding the technique’s applicability. Finally， the review outlines current challenges and prospective directions for the field. Key challenges include the lack of clear selection criteria for carrier materials， potential detrimental effects of immobilization on target structure and function， and the need to balance generality with cost-effectiveness. Future efforts should focus on developing more efficient and targeted immobilization protocols， creating multifunctional composite materials that combine separation， detection， and screening capabilities， fostering interdisciplinary collaborations integrating nanotechnology and bioinformatics， and adhering to green chemistry principles to ensure sustainability. As technological progress continues， bioaffinity fishing is poised to play an increasingly vital role in elucidating the material basis of natural medicine efficacy and accelerating the discovery of new therapeutic agents.

自20世纪80年代至今上市的新药中，50%以上直接或间接来源于天然产物^［1］^，天然产物是药物分子发现的重要源泉，也是国内外新药研究的热点，天然产物活性成分的筛选是助力我国天然药物产业高质量发展的重要推手。

传统天然产物活性成分筛选方法主要依赖化合物的随机搜索，包括筛选药材部位、分离纯化、结构鉴定和生物活性评估等，步骤烦琐且研究周期长^［2，3］^。近年来开展较多的基于细胞筛选系统的高通量筛选技术和高内涵筛选技术拥有快速、灵敏和准确的特点，然而这些技术多为表型筛选（phenotypic method）^［4］^，作用通路/靶标不明确，为后续新药的研究带来一定困难。

为此，直接针对疾病靶标进行活性成分筛选（target-driven method）成为一个较好选择，生物亲和垂钓技术基于配体与配基之间（抗原-抗体、酶-底物、核酸-适配基、激素-受体等）的高度特异性亲和作用，将复杂成分分离与化合物活性筛选相结合，通过进一步与高效液相色谱-质谱联用，实现复杂天然产物体系中活性成分的一步筛选分离分析^［5］^，契合本团队提出的“模型-靶点-分子-机制”中的“靶点-分子”部分，通过对天然产物活性物质基础研究助力新药研发。生物亲和垂钓技术根据分离和分析是否同时进行可分为离线和在线两种模式^［6-8］^，或根据靶标分为酶、受体、核酸、细胞膜、脂质体等不同类型^［9-11］^，均已有前人进行了较好的综述。垂钓材料是生物亲和垂钓技术的关键，其选用的载体材料和固定化方法关系着垂钓过程的准确性和可重复性，因此，本综述以生物亲和垂钓材料为着眼点，分别从载体材料和固定化方法两个维度探讨近3年生物亲和垂钓技术筛选天然产物活性成分的研究进展（图1）。

**图1:**
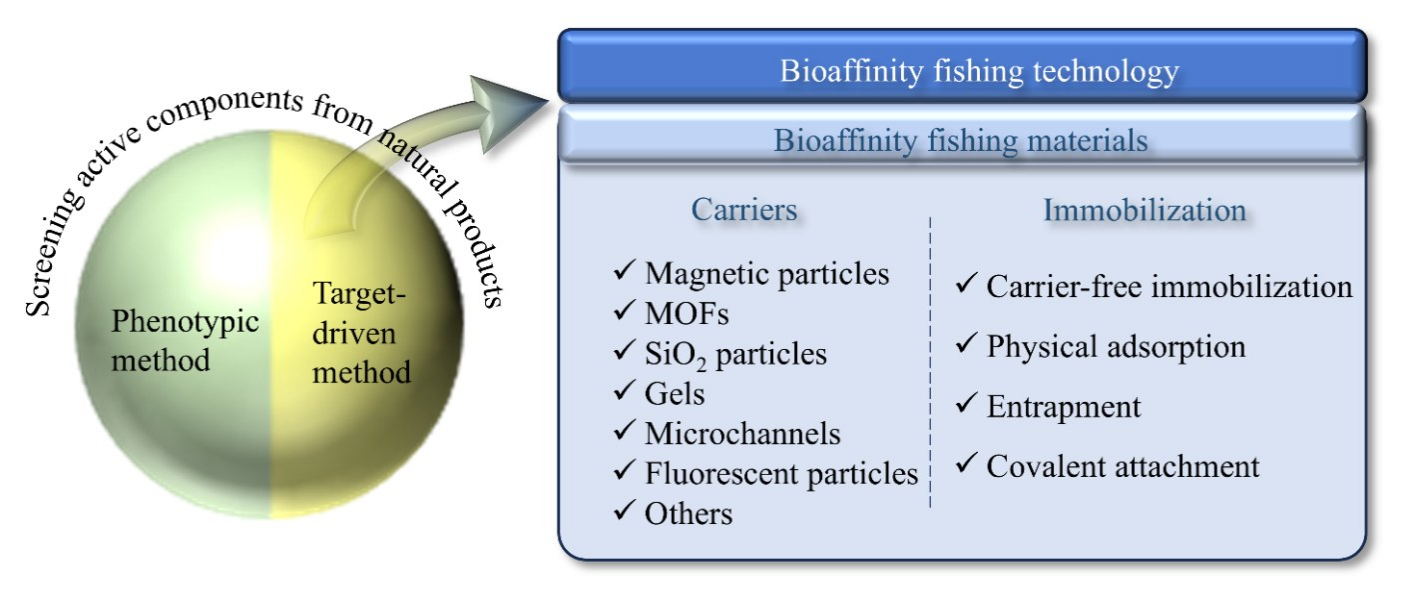
天然产物活性成分筛选技术及不同生物亲和垂钓材料

在此特别说明，以酶分子作为垂钓靶标时，固定化酶微反应器也是一类重要的天然产物活性成分筛选技术（可将其分类于基于生化筛选系统的高通量筛选技术）。该方法通常以酶活性为考察依据，间接评价天然产物中活性成分对靶酶的抑制/促进作用，仅适用于单一化合物或天然产物整体活性的评价，无法从天然产物复杂体系中准确筛选出特异性活性成分化合物，本综述中不予以详细讨论。

## 1 生物亲和垂钓材料的载体

### 1.1 无载体生物亲和垂钓材料

#### 1.1.1 游离靶标

无载体材料中最常规的手段即使用游离靶标亲和捕获目标化合物，然后通过选择孔径合适的超滤膜分析滤液化合物的变化，从而筛选与靶标亲和力较强的活性成分，被称为亲和超滤（affinity ultrafiltration，AUF）技术，一般与液相色谱和质谱联用，最早于1981年由Whitlam等用于评估临床血清结合试验^［12］^。在亲和超滤的实验过程中，一般需要考察提取物和靶蛋白的浓度、超滤膜的材料与孔径、解离溶剂的选择、截留体积、共孵育时间、离心速度和溶液pH等。Wang等^［13］^利用神经氨酸酶与虎杖提取物的亲和超滤，得到了5个潜在治疗甲型流感病毒的化合物。本团队^［14］^通过优化亲和超滤的实验条件，同时使用灭活靶蛋白组进行平行对照实验解决超滤过程中非特异性吸附导致的假阳性或假阴性结果，确保筛选结果的高效性和特异性，从油橄榄果提取物中发现了6个潜在的黄嘌呤氧化酶抑制剂。为体现天然产物多成分多靶标的作用特点，Huang等^［15］^利用环氧合酶-2和透明质酸酶双靶标亲和超滤得到了野菊花中13种皮肤抗炎的活性成分，Zhang等^［16］^利用*α*-葡萄糖苷酶、胰脂肪酶和环氧合酶-2三靶标亲和超滤得到了荷叶中4个活性成分，为从复杂的药用植物提取物中发现功能性活性成分提供了有价值的信息。

除AUF技术外，游离靶标还常与尺寸排阻色谱（size exclusion chromatography，SEC）联用，先将游离靶标和目标提取物共孵育，然后进入SEC-LC-MS联用系统，将两个色谱维度在单个系统中耦合。该技术最早于1997年由Kaur等发明^［17］^，从潜在配体与可溶性大分子靶标的孵育开始，达到平衡后由SEC首先将配体-受体复合物与未结合的化合物分离，然后将早期洗脱的配体-受体复合物离线或在线加载到HPLC反相柱上，受体-配体复合物因流动相中存在有机溶剂而变性，结合的配体被洗脱到质谱仪中用于鉴定。De Soricellis等^［18］^利用该方法筛选得到了墨西哥葱中过氧化物酶体增殖物激活受体*α*和*γ*的激动剂，主要为皂苷类物质，为治疗糖尿病、动脉粥样硬化、血脂异常及相关代谢综合征奠定了物质基础。

#### 1.1.2 固定靶标

无载体的生物垂钓材料中亦有固定化的靶标，即利用交联剂形成蛋白聚集体（酶聚集体）。无载体固定化酶不依赖无活性载体材料，通常通过化学交联法构建，其形成方式主要基于相对分子质量及交联策略的差异。根据形态特征，该方法可分为以下几类：交联溶解酶（cross-linked dissolved enzymes，CLEs）、交联喷雾干燥酶（cross-linked spray dried enzymes，CLSDs）、交联冻干酶（cross-linked enzyme lyophilizates，CLELs）、交联酶晶体（cross-linked enzyme crystals，CLECs）和交联酶聚集体（cross-linked enzyme aggregates，CLEAs）等^［19］^。一般认为，与使用固体载体的方法相比，这些无载体固定化靶标技术可能具有更广泛的适用性，更好的稳定性，更简单的制备方法和更高的体积活性。Jia等^［20］^通过游离酶乙醇沉淀、戊二醛交联、离心洗涤三步法合成了碳酸酐酶CLEAs，从丹参中筛选出5个具有潜在治疗急性高山病的活性成分。CLEAs主要通过提取物孵育后离心取上清液分析化合物成分，而同样作为无载体生物亲和垂钓技术，采用游离碳酸酐酶孵育后超滤无法得到二萜类化合物，因为其在超滤膜上积累而不穿过膜，CLEAs有力地规避了亲和超滤技术活性成分筛选不全面的缺陷。

### 1.2 有载体生物亲和垂钓材料

#### 1.2.1 磁珠

磁性材料的生物相容性好、比表面积大、易于修饰且具有超顺磁性，便于与反应体系利用磁场快速分离，因此被广泛应用于生物亲和垂钓材料的载体。在磁性材料中，最常采用的是已通过羧基/氨基等修饰的商品化磁珠，仅需将目标靶蛋白共价交联即可得到所需的垂钓材料，目前已有大量相关研究，如*α*-葡萄糖苷酶^［21-23］^、环氧合酶-2^［24，25］^、胰脂肪酶^［26］^、黄嘌呤氧化酶^［27］^、单胺氧化酶^［28］^、病毒蛋白（非结构蛋白5（Nsp5）^［29］^、非结构蛋白4（Nsp4）^［30］^、3C样蛋白酶（3CL）^［31］^）、*β*-淀粉样蛋白42（A*β*42）原纤蛋白^［32］^、色氨酸2，3-双加氧酶^［33］^、高表达嘌呤核苷磷酸化酶的结核分枝杆菌^［34］^等。除采用商品化磁珠，也有大量研究通过合成得到合适粒径的Fe_3_O_4_磁性微球或纳米粒，进而修饰目标官能团以固定化靶标蛋白。具体方法如下：1）先以SiO₂包覆，再引入聚多巴胺（polydopamine，PDA）等材料，利用其氨基通过戊二醛与蛋白进行交联固定^［35-37］^；2）直接采用PDA包覆，凭借其强黏附性固定靶标蛋白^［38］^；3）先借助PDA吸附金属离子，进而通过金属离子配位作用固定靶标^［39］^；4）通过修饰羧基，并使用1-乙基-3-（3-二甲基氨基丙基）碳二亚胺（1-ethyl-3-（3-dimethylaminopropyl）carbodiimide，EDC）/*N*-羟基琥珀酰亚胺（*N*-hydroxysuccinimide，NHS）活化共价固定靶标蛋白^［40-44］^。磁性材料因为其优越的性能通常也和其他材料如金属有机框架（metal-organic frameworks，MOFs）、硅基材料、荧光材料等得到多功能复合垂钓材料，具体信息将在对应小节讨论。

#### 1.2.2 MOFs

MOFs是由金属离子或金属簇与有机配体通过配位键自组装形成、具有周期性二维或三维网络结构的多孔晶体材料，具有较大的比表面积和孔隙率、可调节和可设计的结构和官能团、良好的生物相容性，是构筑生物亲和垂钓材料的理想载体。Chen等^［45］^通过合成UiO-66-NH_2_，然后化学交联固定化*α*-葡萄糖苷酶，从芍药种子中垂钓得到9个活性化合物。然而，单独MOFs材料垂钓和再生均需多次离心，操作烦琐且影响靶蛋白活性，磁性的引入可以克服材料回收的限制，因此，多项研究以磁性Fe_3_O_4_纳米粒子为核心，在其表面合成UiO-66-NH_2_^［46，47］^、MIL-101（Cr）-NH_2_^［48］^及ZIF-8-NH_2_^［49］^等，进一步交联目标靶蛋白，利用磁场快速垂钓分离得到潜在活性成分。另一方面，化学交联的蛋白固定化过程中可能存在对靶标的不利构象变化，Hu等^［50］^通过温和条件原位合成ZIF-8并包埋脂肪酶，同时使用胆酸钠作为固定化的改性剂，提高了脂肪酶的稳定性和活性，在胆酸钠增溶的帮助下从莲心叶中筛选得到了9个候选活性化合物。

#### 1.2.3 硅基材料

硅基材料是常用的生物亲和垂钓材料，一般分为离线常用的介孔二氧化硅材料和在线常用的硅胶色谱固定相材料。

介孔二氧化硅具有优异的稳定性、大的表面积和高孔隙率，被广泛应用于固定化酶。通常，介孔硅材料会和磁性纳米粒联合使用，用以提升其使用便捷性。Liu等^［51］^利用戊二醛将吲哚胺2，3-双加氧酶1固定于商业氨基官能化的二氧化硅磁性纳米颗粒上，发现了咖啡中具有潜在抗抑郁的活性成分。Xu等^［52］^通过合成磁性介孔二氧化硅材料并利用戊二醛交联法固定*α*-葡萄糖苷酶，成功地从葛根中筛选出抑制剂葛根素。Liu等^［53］^采用类似方法，借助相同类型的基体材料和固定策略固定*α*-淀粉酶，从五味子中识别出抑制剂5-羟甲基糠醛。在此基础上，Wang等^［54］^进一步对磁性介孔二氧化硅材料进行表面功能化修饰，通过戊二醛交联固定*α*-淀粉酶，构建了聚多巴胺/L-半胱氨酸双功能化复合材料；该改性材料不仅有效防止了纳米颗粒聚集，还为酶固定化提供了更多反应位点，从而显著提升了固定化效果；该方法成功地从库拉索芦荟中筛选出芦荟素A与芦荟素B两种潜在抗糖尿病先导化合物。

硅胶色谱固定相因具有良好的机械强度、耐溶剂性、可控的比表面积和孔径大小、不溶胀和不可压缩性、表面富含易于键合或改性的硅羟基等优点而成为实验室分析检测及中小分子工业分离纯化的最主要填料。在生物亲和色谱固定相中还由于其为惰性材料，可以避免垂钓结果受载体材料的影响。Parsajoo等^［55］^将人髓过氧化物酶通过不同方式固定化于商品化的5 μm HPLC级球形硅胶表面，得到了最优制备方法，为筛选缓解炎症相关症状的化合物提供了新平台。西北大学赵新锋教授团队也在该领域做出了卓有成效的工作。该团队利用卤代烷脱卤素酶标签（Halo-tag）与6-氯己酸的特异性结合生物正交反应构筑了系列受体固定相^［56-61］^，并进一步将其拓展至对抗增生性瘢痕的双靶标垂钓体系中^［62］^；同时，该团队也尝试了其他融合标签类的定向固定化靶标策略，包括组氨酸标签（His-tag）^［63，64］^、化脓性链球菌纤维连接蛋白标签（Spy-tag）^［65］^、CLIP标签（CLIP-tag）^［66］^、表皮生长因子受体ErbB1标签（EGFR-tag）^［67］^、VirD2标签（VirD2-tag）^［68］^、蜡样芽孢杆菌芽孢蛋白标签（CotB1p-tag）^［69］^等；并探索了基于点击化学^［70-72］^和非天然氨基酸插入策略^［73］^的蛋白质定向固定化技术，极大地拓展了受体固定相的构筑策略，具体信息在2.3节“化学交联”模块讨论。

细胞膜色谱是一种以具有活性的细胞膜受体为固定相的生物亲和色谱，此技术利用细胞膜自身的融合作用和硅胶表面硅羟基的吸附作用制备成细胞膜色谱固定相。细胞膜色谱技术自贺浪冲教授于1996年提出以来，已经用于160多种中药或天然药物的活性物质筛选，极大地推动了中药药效物质基础的发现。作为多靶标筛选分析的代表，该团队Liu等^［74］^构筑了5-羟色胺1A受体、5-羟色胺2A受体、多巴胺D1受体和多巴胺D2受体功能化的细胞膜色谱柱，靶向筛选出了远志中9种潜在的多靶标协同抗抑郁活性化合物；该团队Su等^［75］^还将细胞膜色谱拓展至线粒体膜领域，构筑了过表达肉碱棕榈酰基转移酶1A的线粒体膜色谱，筛选出了5种调节脂质代谢的靶标激动剂；近年来，该团队还着重发展了基于His-tag^［76］^、SNAP-tag^［77］^、Halo-tag^［78］^、生物素化标签（Avi-tag）^［79］^和链霉亲和素标签（Strep-tag）^［80］^等生物正交反应的系列蛋白质定向固定化技术，具体信息在“2.3 化学交联”模块讨论。中国人民解放军海军军医大学柴逸峰/陈啸飞团队的Gu等^［81］^发展了3-巯基丙基三甲氧基硅烷改性的细胞膜色谱，得到了黄芩中通过抑制骨髓单个核细胞向破骨细胞分化而具有潜在抗骨质疏松活性的成分；该团队利用此方法进一步构筑了双通道双细胞膜色谱^［82］^和二维过表达/正常细胞膜色谱比较分析系统^［83］^，更好地阐明了多组分和多靶标的中药作用机制。

#### 1.2.4 凝胶

凝胶材料与硅基材料类似，是生物亲和色谱固定相的另一大基体材料，一般采用天然碳水高分子改性材料，与硅胶并列，称为“软胶”。软胶由于亲水性强，生物兼容性好，能减少对生物分子的非特异性吸附等特点，在筛选过程中容易保持靶标蛋白和化合物的生物活性，是生物亲和分离纯化应用历史最悠久、应用最多的基体材料。以琼脂糖为基体是目前商品化最成熟的软胶，Yi等^［84］^基于Spy-tag化学的蛋白质亲和配体定向固定化策略，即以绿色荧光蛋白为捕获蛋白模型，被Spy-tag标记并通过异肽键自连接以特定方向锚定在与Spy接收蛋白偶联的活化琼脂糖表面，筛选了铜绿假单胞菌群体感应通路中的关键酶PqsA抑制剂。然而，琼脂糖基质的机械强度差、易溶胀、复合配基接枝过程复杂、比表面积小，在一定程度上反而限制了亲和配基对抗体的富集分离，人工合成的聚合物凝胶材料近年来受到广泛关注。中国人民解放军海军军医大学柴逸峰和陈啸飞团队的Wang等^［85］^在poly（GMA-co-PEGDA）整体柱上一步合成并通过Strep-tag Ⅱ定向固定膜受体，从丹参提取物中成功筛选出3种潜在的血小板衍生生长因子受体*β*靶向配体。在此基础上，该团队Chen等^［86］^开发了一种基于无细胞蛋白质合成和双（磺基琥珀酰亚胺基）琥珀酸修饰的poly（GMA-co-PEGDA）整体固定相的新型膜受体生物亲和色谱方法，实现了定向可控的免疫检查点受体a第2组家族（NKG2A）的有效合成和固定，筛选出两种新的NKG2A抑制剂黄芩苷和汉黄芩苷，突破了传统亲和固定相的稀有靶标需进行细胞培养、重组蛋白表达等操作的限制。

#### 1.2.5 微通道

基于微通道的生物亲和垂钓材料一般采用在线检测技术，样品用量少且能高通量筛选不同种类的天然产物。其中一类微通道为毛细管柱，Liu等^［87］^将铜绿假单胞菌定植和侵袭的关键靶标LasR利用EDC/NHS共价固定于poly（GMA-co-PEGDA）毛细管柱中，通过与高效液相色谱-质谱联用从黄芩提取物中筛选得到了潜在的LasR抑制剂黄芩素。另一大类是刻于芯片上的微通道，如Qi等^［88］^将PDA修饰于玻璃芯片微通道的内壁上，然后利用其黏附性固定关键靶标单胺氧化酶B，从番红花中得到了治疗帕金森病的潜在活性成分西红花苷I和Ⅱ；Cai等^［89］^利用三相层流芯片建立了一种通过G-四链体从巴豆种子提取物中识别抗肿瘤成分的方法，这种芯片技术集成了目标化合物与提取物复杂成分的分离和有机溶剂提取，简化了预处理过程，减少了试剂用量和操作时间，同时由于微通道是基于扩散分离的温和无膜过滤过程，假阴性结果较少。此外，表面等离子体共振（surface plasmon resonance，SPR）芯片是一类具有成熟商业化操作规程的生物亲和垂钓材料。SPR芯片是通过金属薄膜（通常为金或银）与光相互作用产生的表面等离子体波，生物分子结合至芯片表面时，局部折射率改变，导致共振角偏移，通过实时监测偏移量从而量化靶标与目标化合物的结合/解离过程。商业化的SPR芯片一般包括裸金芯片、羧甲基葡聚糖5（carboxymethylated dextran 5，CM5）芯片、链霉亲和素（streptavidin，SA）芯片、氨三乙酸（nitrilotriacetic acid，NTA）芯片和SpotReady^TM^芯片等，一般用于生物亲和垂钓的是CM5芯片，该芯片在金层表面共价修饰羧甲基葡聚糖层，可以方便地将靶标蛋白通过多种基团固定于芯片微通道，具有高亲水性、低非特异性结合的特点。Su等^［90］^利用固定化环氧合酶-2的CM5芯片从山莨菪中发现了阿托品和法筚枝苷等2种潜在的抗炎药物（图2）。Zhao等^［91］^利用原位切割和排序酶A将*β*2肾上腺素受体固定在裸金芯片上，具有更高的结合亲和力评估准确性。柴逸峰和陈啸飞团队构筑了系列固定促分裂原活化蛋白激酶的CM5芯片^［92］^、固定乳腺癌症耐药蛋白的L1芯片^［93］^、固定刺突蛋白受体结合结构域/血管紧张素转换酶双靶标的CM5芯片^［94］^等，突出了SPR系统在药物筛选中的效率和准确性。

**图2:**
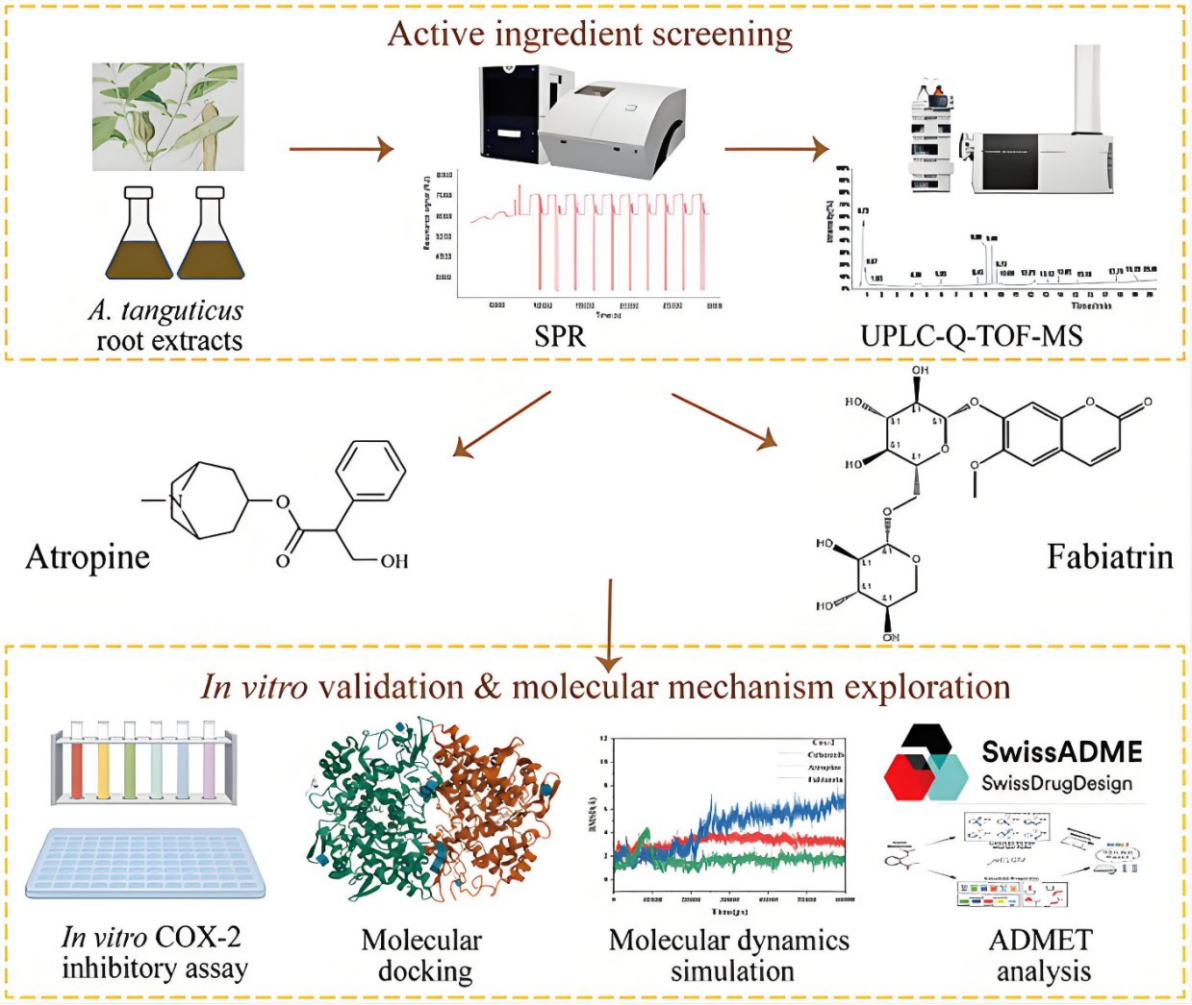
基于SPR芯片的生物亲和垂钓技术^［90］^

#### 1.2.6 荧光材料

荧光材料的加入使生物亲和垂钓与生物医学成像有机结合起来，最早于2018年由Hu等^［95］^提出。他们在CdTe量子点核心外修饰微孔二氧化硅保护性涂层，然后封装于介孔二氧化硅外壳内，将热休克蛋白90共价固定于最外层的介孔外壳上，得到了微孔/介孔纳米多层结构的荧光垂钓材料，从雷公藤提取物中筛选得到了4种抗肿瘤化合物。受此启发，沈阳药科大学赵龙山团队分别采用CuInS_2_/ZnS-Fe_3_O_4_@SiO_2_纳米复合材料^［96］^和氨基修饰的碳量子点^［97］^为荧光垂钓材料，建立了磁荧光配体垂钓和原位成像技术相结合的生物分析平台，分别从龙牙草和黄芩中筛选得到*α*-葡萄糖苷酶抑制剂。与之类似的，Zhao等^［98］^基于多巴胺功能化的碳量子点固定化酪氨酸酶，垂钓后直接进行荧光检测，首先通过已知的天然酪氨酸酶抑制剂槲皮素确认该材料具有可行性和良好的定量性能，然后应用于黄芩和苦参中酪氨酸酶抑制剂的筛选，共发现6种潜在美白活性化合物。Zhang等^［99］^将荧光分子罗丹明B异硫氰酸盐接枝在纤维蛋白原上，结合凝血酶@SiO_2_垂钓材料，显著提高了筛选过程的有效性和准确性。传统生物亲和垂钓材料是通过生物亲和作用筛选材料-酶复合物结合的配体，这些配体不一定具有生物活性，且非特异性结合通常不可避免。此外，在活性测试之前，需要从材料上解吸配体，这受到诸如洗脱溶剂、洗脱时间和pH值等许多因素的影响，从而干扰测定结果。引入荧光材料实时监测垂钓出的化合物的生物活性，可以在一定程度上克服上述缺陷。

#### 1.2.7 其他

石墨烯、碳纳米管等多孔碳材料也常用来做生物亲和垂钓的基体材料。Chi等^［100］^通过β链DNA将过氧化物酶体增殖物激活受体γ固定化在合成的石墨烯磁性材料上，筛选得到了南酸枣中具有治疗冠心病潜力的活性成分。然而，常规石墨烯的层状结构容易聚集，难以在溶液中稳定分散，仅依靠网状石墨烯片作为载体无法实现良好的靶标固定化效率。因此，Zhou等^［101］^合成了定向层状氧化石墨烯垫，其具有良好的可重复性和可回收性，且规则的孔隙结构和低孔曲率可以有效地保证高密度的靶标固定化和待测样品的传质效率，通过在定向层状氧化石墨烯垫上分别固定化表皮生长因子受体高表达和低表达的细胞膜蛋白，发现了紫草中5个表皮生长因子受体抑制剂。Li等^［102］^将酪氨酸酶共价固定于多壁碳纳米管上，发现了白芍中的酪氨酸酶抑制剂；类似地，Wang等^［103］^利用固定化脂肪酶的磁性多壁碳纳米管筛选得到了刺槐中的降脂活性成分；在此基础上，Wang等^［104］^为从菊花中发现脂肪酶抑制剂，利用壳聚糖和离子液体修饰磁性多壁碳纳米管，一方面改善了载体材料的分散性和生物相容性，另一方面利用疏水性离子液体增强垂钓材料的疏水性，从而通过界面相互作用促进脂肪酶盖打开，这种开放构象改善了底物与活性中心的接近度，并促进了固定化脂肪酶的活性和稳定性。

纸基材料是一类价格低廉且使用快捷的垂钓材料基体，Hu等^［105］^通过聚多巴胺修饰的滤纸片黏附固定单胺氧化酶B，从黑果枸杞中发现了4种具有治疗帕金森病潜力的酚酰胺类化合物。Chen等^［106］^更进一步地将可视化活性试纸与垂钓材料结合在一起，与1.2.6节“荧光材料”的设计思路类似，将*α*-葡萄糖苷酶固定在试纸上，模拟*α*-葡萄糖苷酶在小肠壁上的情况，一方面通过生物亲和作用垂钓得到了栓皮栎里*α*-葡萄糖苷酶抑制剂，同时也验证了其具有潜在治疗糖尿病的活性。

此外，也有研究将固相微萃取或膜分离和靶向垂钓技术结合起来，Huang等^［107］^首次采用膜分离结合固相微萃取技术验证了从环境混合物中靶向垂钓筛选乙酰胆碱酯酶活性物质的可行性。该研究一方面将乙酰胆碱酯酶固定在尼龙膜上，另一方面采用直径为100 μm的不锈钢丝制备了固相微萃取纤维，利用硼酸基团与顺式二醇的特异性结合，将乙酰胆碱酯酶固定在固相微萃取纤维上。结果表明，尼龙膜对强疏水性化合物的吸附是不可避免的，膜的非特异性吸附限制了膜分离结合生物亲和垂钓技术对强疏水性化合物的应用，而固相微萃取结合生物亲和垂钓技术能够区分特异性和非特异性吸附的强疏水性化合物，是一种更具有前景的筛选分析材料。

## 2 生物亲和垂钓材料的固定化方法

### 2.1 物理吸附

物理吸附是很多多孔材料固定化靶标的方法，一般为首先合成垂钓材料基体，然后通过超声等辅助手段促进靶标通过物理扩散进入材料孔道，或通过氢键、范德华力、*π-π*相互作用、疏水相互作用、静电相互作用、金属配位、物理黏附等较弱的力使靶标固定在材料上，制备过程不参与化学反应，因此其蛋白结构基本保持不变，活力损失较小，但存在靶点易泄漏和固定化效率低等问题。该方法对材料基体的合成条件无要求，因此垂钓材料的可选择性和可设计性更强。

### 2.2 物理包埋

物理包埋一般用于MOFs材料和凝胶材料，该固定化方法制备过程同样不参与化学交联，靶标活力损失较小，然而，对于凝胶材料，物理包埋一般是将靶标分子与材料基体混合一体成型，存在靶标泄露（凝胶网络过于松散）或传质效率低（凝胶网络过于致密）等问题；MOFs材料一般是将靶标分子首先与有机单体混合，然后加入金属离子，在MOF形成的过程中将靶标原位包埋，不但存在靶标固定化效率低和传质效率低等问题，还存在有机单体或金属离子对靶标构象及活性破坏的风险^［108］^。该方法对材料基体的合成条件要求较高，一般要求常温常压且pH适中，以免对靶标产生不利影响，因此，此类垂钓材料基体类型一般较为单一。

### 2.3 化学交联

化学交联一般利用靶标蛋白中的氨基或羧基等基团，通过加入不同交联剂，采用较温和的方法将蛋白固定于基体材料上。常用交联剂包括利用氨基交联的戊二醛和利用羧基交联的EDC/NHS试剂等。通过共价键设计极大地改善了靶标的固定稳定性，但又存在破坏靶点分子结构或生物活性的风险。该方法同样是先进行材料基体合成，然后固定化靶标，因此对材料基体的合成条件无要求，但要求材料基体修饰有较多可供交联的活性官能团。

在化学交联方法中，有一类重要技术为靶标的定向固定化策略，有助于保持蛋白活性和空间结构的完整性；适用于表达水平极低的蛋白，可以保证固定效果的高效性和可靠性。该策略包括各类基于生物正交反应的大标签如His-tag与Ni-NTA^［63，76］^、Halo-tag与烷基氯^［56-62，78，109］^、Avi-tag与2-亚氨基生物素^［79］^、Spy-tag与Spy接收蛋白^［65，80，84］^、CLIP-tag与苄基胞嘧啶^［66］^、SNAP-tag与苄基鸟嘌呤^［77］^、VirD2-tag与T-DNA^［68］^、EGFR-tag与依鲁替尼^［67］^等，以及基于点击化学和非天然氨基酸插入策略的蛋白质定向固定化技术。生物正交反应具有高选择性（高特异性）、高反应速率、高生物相容性和无副反应等优势，具体而言，反应物只彼此反应，不与细胞内任何天然存在的官能团反应，这是高表达受体靶标无需纯化即可一步固定化的前提；反应即使在很低浓度下也能快速发生，有助于生物体内低浓度靶标的固定化；反应所需的条件与生理条件兼容，并且反应物、催化剂和产物对细胞无毒或毒性极低，保证了靶标的稳定性。在常规单点定向固定方法的基础上，Zhang等^［64］^基于PPAR*γ*与DNA适配体（Apt 2）和His-tag与Ni-NTA的二价亲和结合技术，提供了具有选择性构象的固定化受体方法，可以区分受体的激动剂和拮抗剂。尽管基于大标签的生物正交定向固定化技术已趋于成熟，但在实际应用中易出现目标化合物特征峰的拖尾和加宽并导致严重的不对称峰，导致反应效率低且色谱性能差。赵新锋教授团队的Zuo等^［73］^通过基因编码在受体中掺入非天然氨基酸，基于叠氮炔环加成反应固定化靶标，显著改善了色谱性能，与目标化合物的结合常数比上述传统的大标签高1个数量级，可能是由于结合速率常数的提高和相对相同的解离速率。类似地，该团队^［70，71］^将非天然氨基酸*O*-烯丙基-L-酪氨酸掺入受体中，通过硫烯加成点击反应将融合蛋白固定在硅胶上，不但有效减轻了色谱峰的拖尾现象和特定配体峰的加宽问题，显著提高了色谱性能，还极大地提高了靶标蛋白的稳定性和可重复性，在含有机溶剂流动相的亲和层析分析中具有突出优势。对于内源性蛋白质的定向固定方法，该团队的Zheng等^［72］^通过引入配体导向酰基咪唑化学，实现了活细胞中内源性5-羟色胺转运体的选择性标记和固定化而无需预先纯化，拓展了一步法固定化内源性靶标蛋白的技术平台。

## 3 结论与展望

生物亲和垂钓技术作为一种直接基于靶标的筛选方法，在筛选天然产物活性成分方面显示出了巨大的潜力。随着研究的深入，各种载体材料（如磁珠、金属有机框架、硅基材料等）以及不同的固定化技术（物理吸附、物理包埋、化学交联等）不断被开发和优化，这不仅提升了筛选效率，也为从复杂天然产物中识别潜在药物分子提供了新的思路。

然而，该领域仍面临若干挑战。首先，不同载体材料在实际应用中的选择标准尚不明确，如何根据特定的筛选需求选择最合适的材料仍然是一个问题。其次，固定化过程可能对靶标蛋白的结构和功能产生不利影响，需要更加精细的控制策略以保证靶标的稳定性和活性。此外，虽然多种新型材料和技术的应用拓宽了生物亲和垂钓技术的应用范围，但这些技术的通用性和成本效益仍有待进一步验证。因此，未来的研究应聚焦于以下几个方向：一是探索更高效的靶标固定化策略，减少非特异性结合的同时保持或提高靶标的活性；二是开发多功能复合材料，集成高效筛选、高灵敏度检测和快速分离等功能于一体，实现一站式解决方案；三是加强跨学科合作，结合纳米技术、生物信息学等前沿领域的最新成果，推动生物亲和垂钓技术向更高层次发展；四是注重绿色化学原则的应用，致力于开发环保型、可持续性的材料和技术，降低对环境的影响。总之，随着技术的进步和新方法的出现，生物亲和垂钓技术有望在天然产物活性成分的筛选及新药研发中扮演越来越重要的角色，为药物发现提供强有力的支持。
